# Can a Generative Artificial Intelligence Model Be Used to Create Mass Casualty Incident Simulation Scenarios? A Feasibility Study

**DOI:** 10.3390/healthcare13243184

**Published:** 2025-12-05

**Authors:** Sergio M. Navarro, Angie G. Atkinson, Ege Donagay, Maxwell Jabaay, Sarah Lund, Myung S. Park, Erica A. Loomis, John M. Zietlow, T. N. Diem Vu, Mariela Rivera, Daniel Stephens

**Affiliations:** 1 Division of Trauma, Critical Care, and General Surgery, Department of Surgery, Mayo Clinic, 200 1st St. SW, Rochester, MN 55905, USA; 2 Department of Surgery, The University of Texas Health Science Center at San Antonio, 7703 Floyd Curl Dr, San Antonio, TX 78229, USA; 3 Department of Electrical and Electronics Engineering, Bilkent University, Ankara 06800, Turkey

**Keywords:** trauma centers, multiple trauma, simulation training, generative artificial intelligence

## Abstract

Introduction: Mass casualty incident (MCI) simulation scenarios are developed based on detailed review and planning by multidisciplinary trauma teams. This study aimed to assess the feasibility of using generative artificial intelligence (AI) in developing mass casualty trauma simulation scenarios. The study evaluated a range of mass casualty trauma simulation scenarios generated from a public generative artificial intelligence platform based on publicly available data with a validated objective simulation scoring tool. Methods: Using a large language model (LLM) platform (ChatGPT4, OpenAI, San Francisco, CA, USA), 10 complex MCI trauma simulation scenarios were generated based on publicly available US reported trauma data. Each scenario was evaluated by two Advanced Trauma Life Support (ATLS) certified raters based on the Simulation Scenario Evaluation Tool (SSET), a validated scoring tool out of 100 points. The tool scoring is based on learning objectives, tasks for performance, clinical progression, debriefing criteria, and resources. Two publicly available mass casualty trauma scenarios were similarly evaluated as controls. Revision and recommended feedback was provided for the scenarios, with review time recorded. Post-revision scenarios were evaluated. Interrater reliability was calculated based on Intraclass Correlation Coefficients (2, k) (ICCs). For the scenarios, scores and review times were reported as medians with interquartile range (IQR) as 25th and 75th percentiles. Results: Ten mass casualty trauma simulation scenarios were generated by an LLM, producing a total of 62 simulated patients. The initial LLM-generated scenarios demonstrated a median SSET score of 78.5 (IQR 74–82), substantially lower than the median score of 94 (IQR 93–95) observed in publicly available scenarios. The interrater reliability ICC for the LLM-generated scenarios was 0.965 and 1.00 for publicly available scenarios. Following secondary human revision and iterative refinement, the LLM-generated scenarios improved, achieving a median SSET score of 94 (IQR 93–96) with an interrater reliability ICC of 0.7425. Conclusions: The feasibility study suggests that a structured, collaborative workflow combining LLM-based generation with expert human review may enable a new approach to mass casualty trauma simulation scenario creation. LLMs hold promise as a scalable tool for the development of MCI training materials. However, consistent human oversight, quality assurance processes, and governance frameworks remain essential to ensure clinical accuracy, safety, and educational value.

## 1. Introduction

The development and execution of mass casualty incident (MCI) simulation scenarios are critical components of emergency preparedness and response training for multidisciplinary trauma teams [1]. Traditionally, these scenarios are crafted through meticulous planning and review, drawing upon the expertise and insights of seasoned clinicians and emergency response coordinators [2,3]. The process, while invaluable, is notably labor-intensive and heavily reliant on manual efforts, often constrained by the availability of experienced personnel and resources [3,4]. Furthermore, the scenarios developed through these conventional means may not always reflect the statistical reality of mass casualty incidents both within the United States and globally, potentially limiting their relevance and effectiveness in preparing medical teams for the diverse challenges posed by actual mass casualty events [5].

Simulation-based training for MCI response has traditionally relied on labor-intensive scenario development by experienced clinicians and emergency coordinators, but these conventional approaches may not fully reflect the statistical realities or operational complexities of actual incidents, limiting their relevance and effectiveness [6,7,8]. Disaster medical response presents unique challenges including high victim numbers, initial disorder, resource shortages, and the need for multidisciplinary teamwork—requiring a system that can rapidly prioritize and optimize resources to minimize loss of life and suffering [9,10]. The field remains relatively new, with much of the current knowledge based on descriptive studies and expert opinion rather than experimental evidence [9,11]. Recent advances in simulation, including virtual reality, smartphone-based, and hybrid models, offer promising, scalable, and more realistic training modalities that can better address the dynamic and resource-constrained nature of disaster response, while also facilitating iterative improvement of preparedness plans [12,13,14,15]. However, ongoing research and innovation are needed to strengthen the evidence base and ensure that simulation scenarios accurately support the goals of disaster medical response systems [15].

In this context, the exploration of innovative approaches to enhance the efficiency, flexibility, and evidence-based nature of trauma simulation scenario development is imperative. One such promising avenue is the application of generative artificial intelligence (AI) models.

This study aims to assess the feasibility of leveraging generative AI to create a broad spectrum of mass casualty trauma simulation scenarios based on publicly available data on MCIs.

By using a major large language model platform’s capability, this research endeavors to assess the feasibility of using artificial intelligence and combined intelligence to generate complex interdisciplinary trauma simulation scenarios grounded in publicly available data on the most prevalent mass casualty events.

## 2. Materials and Methods

This study employed a generative large language model (LLM) platform, ChatGPT4 (OpenAI, San Francisco, CA, USA), to innovate the process of creating mass casualty trauma simulation scenarios as a feasibility study. This approach aimed to introduce a novel method that could potentially streamline the development of simulation scenarios that are grounded in statistical evidence of mass casualty events. The TRIPOD-LLM checklist was adhered to (https://tripod-llm.vercel.app/, accessed on 1 September 2025), with a checklist available in Supplementary File S1. The study used publicly available databases and was exempted from review by the Institutional Review Board.

### 2.1. Data and Analytical Methods

The development process involved three main stages, (1) Database creation, (2) Custom LLM development, and (3) Integration, followed by post hoc deployment of a research tool available for usability testing.

#### 2.1.1. Database Creation

A database was implemented to store structured input prompts, generated outputs, metadata, and evaluation data. This database organized trauma event data drawn from national repositories such as the National Trauma Data Bank (NTDB), National Electronic Injury Surveillance System (NEISS), National Fire Protection Agency (NFPA), United States Coast Guard (USCG), and National Traffic and National Transportation Safety Board (NTSB). Data was parsed and organized into a structured set of tables and files into an aggregate data repository including the most common trauma and possible MCI trauma scenarios.

#### 2.1.2. Custom LLM Development

A custom GPT-4-powered model, TraumaSurgGPT, was created using OpenAI’s GPT platform. It was specifically designed to generate high-fidelity, educational trauma simulation scenarios. The model was tuned via system-level instructions to ensure realism, appropriate clinical terminology, and educational structure. Instructions emphasized medical accuracy, inclusion of trauma procedures and resources, and tailoring by user-defined needs (e.g., injury type, training level).

The LLM was instructed to base these scenarios on patterns and characteristics common to mass casualty incidents, with a specific focus on those most frequently encountered in the U.S., based on underlying database inputs from the aggregate data repository.

**Figure 1:**
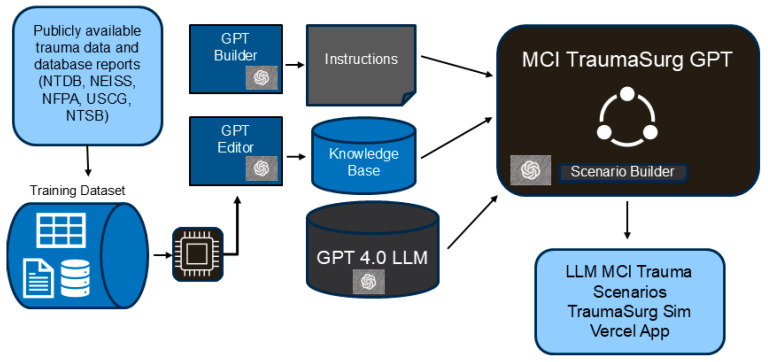
Diagram of LLM MCI trauma model and scenario development. Summary of process for Generative Pre-trained Transformer (GPT)-based large language model (LLM) mass casualty incident (MCI) scenario development. National Trauma Data Bank (NTDB), National Electronic Injury Surveillance System (NEISS), National Fire Protection Agency (NFPA), United States Coast Guard (USCG), and National Traffic and National Transportation Safety Board (NTSB).

The database data was aggregated and collected into an LLM based on these inputs and combined with the Generative Pre-trained Transformer (GPT) editor and GPT builder to construct an MCI trauma-specific GPT that generated MCI trauma scenarios (Figure 1).

#### 2.1.3. Integration

An OpenAI GPT-4 Application Programming Interface (API) key was used to enable connection between the database and the GPT-4 large language model. The model generated mass casualty trauma simulation scenarios based on structured prompt inputs referencing the data repository. The cost of using the GPT-4 API was based on token usage, at a rate of approximately USD 0.03 per 1000 tokens (~750 words). Each scenario used an average of 1400 tokens (approx. USD 0.04 per scenario), generating around 1000 words per simulation.

##### LLM Architecture, Hyperparameters, and Prompting

The GPT-4 architecture from OpenAI was used with model system-prompt and template prompting without gradient-based fine-tuning performed, based on the database reference based on parameterization. Simulation scenarios were further optimized through prompt engineering techniques, using structured templates with embedded statistical summaries from national trauma registries. Prompt hyperparameters were optimized for temperature, maximum token count, frequency penalty and presence penalty, and seed. Prompts instructed the model to simulate trauma scenarios with multiple victims based on real-world situations and included explicit instructions to generate detailed scene narratives, patient vitals, interventions, and debriefing content. The prompts used for parameterization of the model as well as training are available in Supplementary Materials (Supplemental File S2). The GPT-4 model used was aligned with OpenAI’s Reinforcement Learning from Human Feedback (RLHF), with internal alignment goals emphasizing helpfulness, honesty, and harmlessness [16]. No gradient-based fine-tuning or retraining was performed.

- **Compute Resources**

Scenario generation occurred through the OpenAI GPT-4 API, with each scenario using approximately ~1400 tokens, ~USD 0.04, with <60 s per scenario for compute. Computing was handled via OpenAI’s infrastructure; metrics such as FLOPs and hardware specifications were proprietary. Total compute time for all scenarios was <1 h. Supabase operated under free-tier hosting with no additional cost.

- **Scenario Generation**

A total of ten complex, interdisciplinary trauma simulation scenarios were generated using ChatGPT4 in combination with national database data and reporting, with a total of 62 simulated patients created.

Scenarios varied in nature, ranging from a major motor vehicle collision to a building fire to a mass shooting incident (Supplementary Materials and Table 1). This directive aimed to ensure that the generated scenarios were not only diverse but also relevant to the current trauma landscape, enhancing their educational value for multidisciplinary trauma teams.

- **LLM Output**

Each scenario output included 67 components. These components for each scenario included details for supporting materials, resources, case stem, physical exam, vitals, and interventions for each individual patient involved in the scenario, as well as debriefing materials with supporting references.

- **Summarization and Outcome Assessment**

Each generated scenario output was evaluated by two raters, both of whom were certified in Advanced Trauma Life Support (ATLS).

- **Quantitative Review**

Each reviewer independently assessed the scenarios using a structured rubric, the Simulation Scenario Evaluation Tool (SSET), a rigorously validated instrument designed to assess various dimensions of simulation scenarios [17]. The SSET emphasizes ATLS-based learning objectives, which are pivotal in trauma management training, along with providing points for completion of performance tasks, scenario consistency, debriefing criteria, and resource requirements. An example of the SSET grading rubric is available in Supplemental File S1. This comprehensive validated framework for simulation ensured an adequate assessment of the educational and operational feasibility of each AI-generated scenario. Reviewers were blinded to one another’s assessments. Inter-assessor agreement of SSET scores for each scenario was measured using Intraclass Correlation Coefficients (ICCs) as a statistical measure used to assess the reliability or agreement of measurements made by multiple raters, observers, or instruments evaluating the same subjects [18].

In addition to the AI-generated scenarios, two publicly available mass casualty trauma scenarios available in the public domain were evaluated using the same methodology [19,20]. This comparative analysis aimed to benchmark the AI-generated scenarios against existing scenarios to gauge their relative effectiveness and realism.

- **Subjective Review**

Following the structured evaluation, each scenario underwent a subjective review process focusing on accuracy, fidelity, and clinical utility. This review allowed for the assessment of each scenario’s realism and applicability to real-world mass casualty incidents. Feedback gathered during this phase was instrumental in identifying areas for refinement, ensuring that the final scenarios were not only statistically grounded but also clinically sound and practically deployable.

- **Revision Process**

Scenarios that received significant feedback during the subjective review phase were subjected to a secondary revision. This iterative process involved revising the scenarios based on the evaluators’ inputs, with a focus on enhancing the scenarios. Total time taken by evaluators for the review of each scenario was recorded, in addition to notation of whether any scenarios needed to be discarded due to hallucinations. The revised scenarios were then re-evaluated to ensure that the modifications effectively addressed the initial concerns, thereby optimizing the scenarios for educational use.

- **Statistical Analysis**

Pre-revision, control, and post-revision scenario SSETs were characterized. Scores are reported as medians with IQR as 25th and 75th percentiles. Interrater reliability was calculated based on ICCs, with two-way random, absolute agreement ICCs ((2,k) with 95% CIs for scenarios using R Statistical Package software (R Foundation for Statistical Computing, Version 4.5.1., Vienna, Austria). Based on the 95% confident interval of the ICC (2,1) estimate, values less than 0.5, between 0.5 and 0.75, between 0.75 and 0.9, and greater than 0.90 were characterized as poor, moderate, good, and excellent reliability [18].

- **Deployment of Research Tool**

Following scenario generation and expert analysis, a user-facing interface was deployed on a Vercel based framework (San Francisco, CA, USA), enabling broader researcher access to the simulation bank. This static web platform was designed for submitting evaluation prompts and for researchers to review individually generated trauma scenarios for annotating or scoring simulations for training, validation, and further development.

Vercel was used under a free-tier license, incurring no infrastructure cost. The deployment supported reproducibility and ongoing educational research on AI-generated medical simulations. Details for access to the tool are available in the Data Availability Section.

## 3. Results

- Scenario Scoring

This study reviewed ten mass casualty trauma simulation scenarios generated by a generative AI model, encompassing 62 simulated patients. The evaluation of these scenarios, based on the SSET, revealed a median score of 78.5 (IQR 74–82) for the AI-generated scenarios.

**Figure 2:**
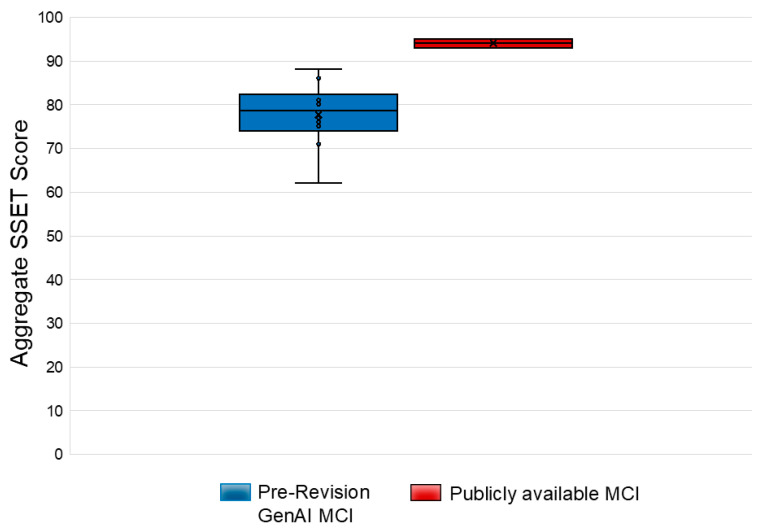
Comparison of total pre-revision and control SSET scores per MCI scenario.

In contrast, scenarios that were publicly available and traditionally used for mass casualty trauma simulations scored higher, with a median SSET score of 94 (IQR 93–95), found in Figure 2.

- Interrater reliability

The interrater reliability, assessed through ICC (2,1) estimates, indicated substantial agreement for the AI-generated scenarios (0.965) and moderate agreement for the publicly available scenarios (0.667). This discrepancy underscores that while there was variability between the scoring of scenarios, there was still adequate reliability between the raters.

- Revision process

Significant feedback, focusing on enhancing accuracy, consistency, and clinical utility, was provided for the LLM-generated scenarios, with a median review time per scenario estimated by each of the reviewers at 25 min [15–33 min] per scenario. Such feedback was instrumental in refining these scenarios, leading to a secondary revision phase. Examples of requested feedback of an excerpt of a single MCI scenario, including 6 selected components of the total 64 components for that specific scenario, with revisions, are found in Table 1. No scenarios needed to be discarded due to hallucinations.

- Post-Revision Scoring

**Figure 3:**
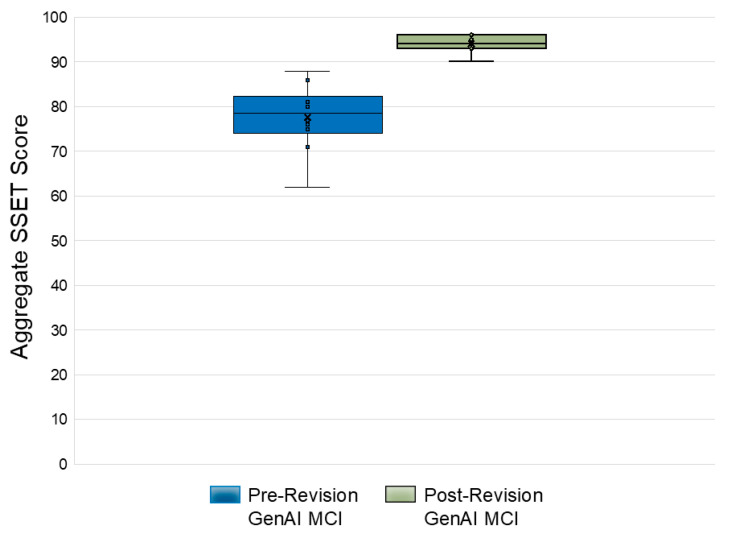
Comparison of total pre-revision and post-revision SSET scores per MCI scenario.

Upon re-evaluation post-revision, the AI-generated scenarios exhibited a marked improvement, achieving a median SSET score of 94 (IQR 93–96) across scenarios, compared to the scores of existing, publicly available mass casualty incident (MCI) scenarios. A comparative scoring of each scenario is found in Figure 3. The interrater reliability, assessed through ICC, indicated moderate reliability for the post-revision scenarios (0.7425).

Simulation Scenario Evaluation Tool (SSET) scores reported for pre-revision generative AI and publicly available mass casualty incident (MCI) scenarios (averaged across raters).

Mean Simulation Scenario Evaluation Tool (SSET) scores reported for pre-revision and post-revision mass casualty incident (MCI) scenarios (averaged across raters).

## 4. Discussion

The integration of generative AI in the creation of MCI simulation scenarios marks an advancement in trauma training methodologies. The manual nature of MCI simulation scenarios results in a limited number of available public scenarios, which may not be based on evidence of data. This feasibility study evaluating the process along with face and content validity of AI-generated scenarios explored the use of a generative LLM platform, ChatGPT4, to produce complex, interdisciplinary trauma simulation scenarios. It represents a novel approach that aims to enhance efficiency, relevance, and adaptability of trauma training exercises. While only preliminary and not a fully powered comparative study, when refined through human feedback with short time investment, the revised AI-generated scenario SSET scores may mean that AI-generated scenarios can achieve a level of quality similar to traditional, manually developed scenarios.

This finding hints at the potential of AI to complement and streamline the scenario development process, aligning it more closely to be grounded from a statistical perspective yet open to the diverse challenges of MCIs. Furthermore, the relatively higher scores seen with the iteration of scenarios point to the ability of LLMs to improve over time and with feedback; this potential improvement will be further evaluated with a comparative analysis and explored in future work. Utilizing LLMs to create scenarios could potentially provide time savings for educators when researching and crafting practical, unique scenarios independently; however, further work with a prospective trial could be constructed to compare the time and work effort required for the development of a human-made scenario with simultaneous development of a combined intelligence-related scenario.

LLM usage for the development of complex outputs in medicine has been increasingly explored in surgery [21]. However, existing studies have shown the limitations of LLM outputs in medicine [22,23]. Accuracy, predictability, and explainability are limiting constraints of LLM outputs [23,24]. However, this study serves primarily as a feasibility assessment. It lays the groundwork for more comprehensive research. We show the non-inferiority of LLM outputs in a combined intelligence format to develop MCI trauma simulation scenarios. Future endeavors may evolve into pilot studies involving a broader range of evaluators, including ATLS-certified trauma surgeons and multidisciplinary trauma team members, to provide a more diverse and expert assessment of the generated scenarios. Incorporating feedback from a wider array of clinical professionals could enhance the scenarios’ realism, clinical utility, and educational value. 

Despite the encouraging performance of the LLM-generated scenarios when assessed using the SSET, it is important to recognize the tool’s inherent limitations in the context of MCI simulation. The SSET was designed to evaluate the overall structural and educational quality of simulation scenarios, focusing on domains such as realism, clarity of objectives, and learner engagement. However, it does not evaluate MCI-specific operational constructs, including triage accuracy, resource utilization, surge capacity, and nonlinear team dynamics under crisis conditions. This gap underscores a broader limitation in the field: no widely accepted, validated checklist currently exists for assessing MCI scenario content, particularly regarding triage frameworks, resource logistics, or complex team flow. While recent tools—such as Schulz et al.’s prehospital disaster response self-assessment instrument [25], Debacker et al.’s SIMEDIS discrete-event modeling system [9], and Regev et al.’s adaptation of the Trauma Non-Technical Skills Scale (T-NOTECHS) for multicasualty trauma care [26]—represent important progress in this area, they remain fragmented and lack a unified, validated structure for scenario scoring. Consequently, our reliance on the SSET provides insight into general scenario quality but does not capture operational fidelity pertinent to MCI-specific response. This highlights a need for future research to develop and validate content for MCI-focused scenario evaluation frameworks, ideally integrating both content validity and operational realism through both expert consensus and empirical testing.

Moreover, this study acknowledges the importance of combined intelligence, where AI’s innovative capacity is balanced with human expertise and oversight [27]. The significant improvements in SSET scores following human-guided revisions of the AI-generated scenarios underscore the need for a symbiotic relationship between AI-generated content and human clinical knowledge. This collaborative approach ensures that the scenarios are not only statistically and algorithmically sound but also clinically relevant and tailored to the specific needs and contexts of different institutions and trauma centers.

Limitations of this study include its preliminary nature, the relatively small number of scenarios, and the raters involved. Furthermore, bias was not explicitly evaluated as part of the scenario review. Only two publicly available MCI scenarios were graded in this feasibility study, limiting the number of scenarios graded. The reliance on ATLS-certified raters for evaluating scenarios, rather than ATLS instructors or seasoned trauma surgeons, may also impact the depth of feedback and scenario refinement, and increasing the number of raters would decrease risk of rater bias. As nationally publicly available databases and dataset-based reporting was used as inputs by the LLM, use of the tool should be limited outside of the U.S. Furthermore, use of U.S.-centric database inputs may limit applicability of data and scenarios outside the U.S. Further work could expand to include global trauma databases, including the data published by the International Road Traffic and Accident Database (IRTAD) and the Emergency Events Database (EM-DAT). The database inputs were restricted to the timeframes for which publicly available data existed at the time of model development. While no independent validation of the databases’ accuracy or potential reporting biases was conducted, these sources, including the NTBD and NSTB, adhere to established internal review and reporting standards, which lend a baseline level of credibility to their data.

Further work in this domain could include an evaluation phase evaluating for bias of data inputs and a post-evaluation phase evaluating the scenarios for potential bias. Future studies, including a pilot study, could expand the pool of raters and involve a more diverse group of trauma care professionals to enrich the scenario development and evaluation process. Rater bias along with data input and scenario bias was not fully evaluated, and future work will use independent, blinded raters and randomized scenario ordering. Additionally, further prompting and feedback provided to a test LLM via manual testers and researchers or by clinical agents may increase the clinical accuracy of such models [27].

Additional work may include but is not limited to broader distribution of the scenarios publicly after a pilot study, enabling the utilization of these scenarios by institutional trauma training teams as a table-top exercise. Evaluation of a larger set of human-authored MCI scenarios from multiple repositories would support comparative benchmarking in a comparative study. Separately, gamification of these scenarios into individual and team-based scenarios may be feasible.

## 5. Conclusions

In summary, this study demonstrates the feasibility of utilizing generative AI to create MCI simulation scenarios, with the potential to make trauma training more efficient, flexible, and evidence-based. The positive outcomes of AI–human collaboration in this study pave the way for further research, emphasizing the need for continued innovation in trauma education and preparedness.

As we move forward, it will be crucial to maintain a balanced approach that leverages the strengths of both AI and human expertise to optimize trauma training and ultimately enhance patient care in MCI.

## Figures and Tables

**Table 1:** Review of excerpt of MCI trauma scenario 6: mass shooting at shopping center.

Prior to Review	Post Review (Changes in Italics)	SSET Domain	How Review Improved the Score
**Initial Assessment:** This scenario is designed to simulate a traumatic event involving multiple gunshot wound victims. It focuses on rapid trauma assessment, management, and surgical consultation for gunshot wounds.	**Initial Assessment:** This scenario is designed to simulate a traumatic event involving multiple gunshot wound victims. It focuses on rapid trauma assessment, management, and surgical consultation for gunshot wounds.	Learning Objectives (Element 1)	No change
**Background:** Gunshot wounds can lead to significant morbidity and mortality depending on the location and trajectory. Immediate assessment, intervention, and surgical consultation are crucial.	**Background:** Gunshot wounds can lead to significant morbidity and mortality depending on the location and trajectory. Immediate assessment, intervention, and surgical consultation are crucial.	Learning Objectives (Element 1)	No change
**Initial Presentation:** Six patients are brought in by EMS after an active shooter incident.	**Initial Presentation:** Six patients are brought in by EMS after an active shooter incident.	Clinical Context (Element 2)	No change
**Scenario Unfolding**: The team must stabilize, diagnose, and manage each patient’s injuries. Some patients may need immediate surgical intervention, while others may require imaging and observation.	**Scenario Unfolding:** The team must stabilize, diagnose, and manage each patient’s injuries. Some patients may need immediate surgical intervention, while others may require imaging and observation.	Clinical Actions (Element 3)	No change
**Actions:** Immediate trauma assessment, imaging, and surgical consultation.	**Actions:** Immediate trauma assessment, imaging, and surgical consultation.	Clinical Actions (Element 3)	No change
**Minute-by-Minute Review:** 0–1 min: Arrival of patients. Briefing by EMS on the nature of injuries for a quick overview. 1–3 min: Rapid primary survey for all six patients. Immediate interventions such as cervical spine immobilization and supplemental oxygen provision. Two trauma bays should be prepped to receive the most critical patients. 3–5 min: Airway assessment and management for patients with altered mental status or respiratory distress. Initiation of two large-bore IVs for fluid and blood products as needed. 5–7 min: Rapid assessment of chest wounds. Needle decompression for suspected tension pneumothorax. Emergency chest tube insertion for patients with significant hemothorax or persistent air leak. 7–10 min: Secondary survey with a specific focus on gunshot wound trajectories. FAST (Focused Assessment with Sonography for Trauma) exams for patients with abdominal gunshot wounds. Direct pressure on thigh wound and assessment of distal pulses. 10–12 min: X-ray ordered for chest gunshot wound victims to ascertain bullet trajectory and organ involvement. CT scan ordered for abdominal gunshot wound victims after FAST exams. 12–15 min: Communication with surgical teams (general surgery for abdominal wounds, vascular surgery for thigh wound, and cardiothoracic surgery for chest wounds). 15–17 min: Blood products (PRBCs, FFP, Platelets) being administered as required. Continued monitoring and reassessment of all patients. 17–20 min: Results from imaging return, guiding surgical and interventional decisions. Decision made on immediate surgical interventions vs. observation vs. non-operative management. 20–25 min: Continued stabilization, preparation for surgery for those who need it. Wound dressing and pain management for patients not going immediately to the OR. 25–30 min: Handoff to surgical teams or ICU/ward teams for further management. Debriefing of the initial trauma team.	**Minute-by-Minute Review:** 0–1 min: Arrival of patients. Briefing by EMS on the nature of injuries for a quick overview. 1–3 min: Rapid primary survey for all six patients. Immediate interventions such as cervical spine immobilization and supplemental oxygen provision. Two trauma bays should be prepped to receive the most critical patients. 3–5 min: Airway assessment and management for patients with altered mental status or respiratory distress. Initiation of two large-bore IVs for fluid and blood products as needed. *Mass Transfusion protocol (MTP) activation as needed for those with hemodynamic instability.* 5–7 min: Rapid assessment of chest wounds. Needle decompression for suspected tension pneumothorax. Emergency chest tube insertion for patients with significant hemothorax or persistent air leak. 7–10 min: Secondary survey with a specific focus on gunshot wound trajectories. FAST (Focused Assessment with Sonography for Trauma) exams for patients with and without thoracic abdominal gunshot wounds. *Tourniquet takedown as needed with assessment of thigh wounds.* Direct pressure on thigh wound and assessment of distal pulses. 10–12 min: X-ray ordered for chest gunshot wound victims to ascertain bullet trajectory and organ involvement. CT scan ordered for abdominal gunshot wound victims after negative FAST exams. 12–15 min: Communication with surgical teams (general surgery for abdominal wounds, vascular surgery for thigh wound, *and immediate operating room (OR) for positive FAST exam in the thoracic cavity with hemodynamic instability. Transport to CT for imaging if stable.* 15–17 min: Blood products (Packed Red Blood Cells (PRBCs), Fresh Frozen Plasma (FFP), Platelets) being administered as required. Continued monitoring and reassessment of all patients. 17–20 min: Results from imaging return, guiding surgical and interventional decisions. Decision made on immediate surgical interventions vs. observation vs. non-operative management. 20–25 min: Continued stabilization, preparation for surgery for those who need it. Wound dressing and pain management for patients not going immediately to the OR. 25–30 min: Handoff to surgical teams or ICU/ward teams for further management. Debriefing of the initial trauma team.	Patient States (Element 4)	Improved Clinical Appropriate Case Management (Element 4, Item 13), Improved Effectiveness of Meeting Learning Objectives (Element 4, Item 15).

## Data Availability

The GitHub code for model development and deployment is available at https://github.com/mitflyboy13/trauma-surg-llm-scenarios, accessed on 1 September 2025. The tool for generation of the model is available to be used for research purposes only at https://traumasurggpt.vercel.app/, accessed on 1 September 2025.

## Supplementary Materials

The following supporting information can be downloaded at https://www.mdpi.com/article/10.3390/healthcare13243184/s1, Table S1: Scenario summary; Table S2: Scenarios Materials; Table S3: Scenarios SSET Scoring and Review Time; Table S4: Databases Summary; Figure S1: Simulation Scenario Evaluation Tool (SSET) report, Summary of the Simulation Scenario Evaluation Tool (SSET) scoring rubric; File S1: TRIPOD-LLM checklist file; File S2: GPT creation model training instructions Refs. [28,29,30] are cited in File S1.

## Abbreviations

The following abbreviations are used in this manuscript:

AI	Artificial Intelligence
API	Application Programming Interface
ED	Emergency Department
EMS	Emergency Medical Services
FAST	Focused Assessment with Sonography for Trauma
GCS	Glasgow Coma Scale
GenAI	Generative Artificial Intelligence
GPT	Generative Pre-trained Transformer
HR	Heart Rate
IO	Intraosseous
LLM	Large Language Model
MAP	Mean Arterial Pressure
MCI	Mass Casualty Incident
NEISS	National Electronic Injury Surveillance System
NFPA	National Fire Protection Agency
NHTSA	National Highway Traffic Safety Administration
NTDB	National Trauma Data Bank
NTSB	National Traffic and National Transportation Safety Board
RLHF	Reinforcement Learning from Human Feedback
RR	Respiratory Rate
SBP	Systolic Blood Pressure
SSET	Surgical Simulation Evaluation Tool
US	United States
USCG	United States Coast Guard
