# Supplementary material for: Can a Generative Artificial Intelligence Model Be Used to Create Mass Casualty Incident Simulation Scenarios? A Feasibility Study
Source: Healthcare (Basel). 2025 Dec 5;13(24):3184. doi: 10.3390/healthcare13243184 (PMC12732669; doi:10.3390/healthcare13243184)
Supplement: Supplementary file 1 [file healthcare-13-03184-s001.zip › File S1.pdf]

TRIPOD+AI checklist for the reporting of prediction model studies

Section/ Topic	Item No	Development/ Evaluation*	Checklist Item	Reported on Page Number/Line Number	Reported on Section/Paragraph
**Title**					
Title	1	D;E	Identify the study as developing or evaluating the performance of a multivariable prediction model, the target population, and the outcome to be predicted	page 1 Line1-3	tittle
**Abstract**					
Abstract	2	D;E	See TRIPOD+AI for Abstracts checklist	page 1 Line5-34	Abstract Paragraph1-4
**Introduction**					
Background	3a	D;E	Explain the healthcare context (including whether diagnostic or prognostic) and rationale for developing or evaluating the prediction model, including references to existing models	page 1-2 Line36-68	IntroductionParagraph1-3
	3b	D;E	Describe the target population and the intended purpose of the prediction model in the context of the care pathway, including its intended users (eg, healthcare professionals, patients, public)	page1-2 Line36-68	IntroductionParagraph1-3
	3c	D;E	Describe any known health inequalities between sociodemographic groups	page 1-2Line36-68	IntroductionParagraph1-3
Objectives	4	D;E	Specify the study objectives, including whether the study describes the development or validation of a prediction model (or both)	page 2Line64-68	IntroductionParagraph 3
**Methods**					
Data	5a	D;E	Describe the sources of data separately for the development and evaluation datasets (eg, randomised trial, cohort, routine care or registry data), the rationale for using these data, and representativeness of the data	page 2 Line71-77	Eligibility criteria and patie nts Paragraph1
	5b	D;E	Specify the dates of the collected participant data, including start and end of participant accrual; and, if applicable, end of follow-up	page 2 Line71-77	Eligibility criteria and patie nts Paragraph1
Participants	6a	D;E	Specify key elements of the study setting (eg, primary care, secondary care, general population) including the number and location of centres	page 2-3 Line78-93	Eligibility criteria and patie nts Paragraph2
	6b	D;E	Describe the eligibility criteria for study participants	page 2-3 Line78-93	[illegible] ntsParagraph2
	6c	D;E	Give details of any treatments received, and how they were handled during model development or evaluation, if relevant	page 2-3 Line78-93	Eligibility criteria and patie ntsParagraph2

Data preparation	7	D;E	Describe any data pre-processing and quality checking, including whether this was similar across relevant sociodemographic groups	page 3 Line102-110	[illegible] and Image Preprocessing P aragraph1
Outcome	8a	D;E	Clearly define the outcome that is being predicted and the time horizon, including how and when assessed, the rationale for choosing this outcome, and whether the method of outcome assessment is consistent across sociodemographic groups	page 3 Line94-101	Eligibility criteria and patie nts Paragraph3
	8b	D;E	If outcome assessment requires subjective interpretation, describe the qualifications and demographic characteristics of the outcome assessors	page4 Line145-155	Plaque Model Construction Paragraph1
	8c	D;E	Report any actions to blind assessment of the outcome to be predicted	None	None
Predictors	9a	D	Describe the choice of initial predictors (eg, literature, previous models, all available predictors) and any pre-selection of predictors before model building	page 2 Line45-54	IntroductionParagraph2
	9b	D;E	Clearly define all predictors, including how and when they were measured (and any actions to blind assessment of predictors for the outcome and other predictors)	page3-4 Line119-155	[illegible] Model and Plaque ModelP aragraph1
	9c	D;E	If predictor measurement requires subjective interpretation, describe the qualifications and demographic characteristics of the predictor assessors	page 4Line145-155	Plaque Model Construction Paragraph1
Sample size	10	D;E	Explain how the study size was arrived at (separately for development and evaluation), and justify that the study size was sufficient to answer the research question. Include details of any sample size calculation	None	None
Missing data	11	D;E	Describe how missing data were handled. Provide reasons for omitting any data	None missing data	None
Analytical methods	12a	D	Describe how the data were used (eg, for development and evaluation of model performance) in the analysis, including whether the data were partitioned, considering any sample size requirements	page2 Line71-77	Eligibility criteria and patie nts Paragraph1
	12b	D	Depending on the type of model, describe how predictors were handled in the analyses (functional form, rescaling, transformation, or any standardisation)	page4-5 Line156-197	Network Details Paragraph 1
	12c	D	Specify the type of model, rationale[†], all model building steps, including any hyperparameter tuning, and method for internal validation	page4-5 Line156-197	Network Details Paragraph 1
	12d	D;E	Describe if and how any heterogeneity in estimates of model parameter values and model performance was handled and quantified across clusters (eg, hospitals, countries). See TRIPOD-Cluster for additional considerations[‡]	page4Line157-165	Network Details Paragraph 1
	12e	D;E	Specify all measures and plots used (and their rationale) to evaluate model performance (eg, discrimination, calibration, clinical utility) and, if relevant, to compare multiple models	page5 Line198-208	[illegible] n of the Prediction Model P aragraph1

	12f	E	Describe any model updating (eg, recalibration) arising from the model evaluation, either overall or for particular sociodemographic groups or settings	page4 Line157-165	Network DetailsParagraph 1
	12g	E	For model evaluation, describe how the model predictions were calculated (eg, formula, code, object, application programming interface)	Supplementary Materials	Supplementary Materials
Class imbalance	13	D;E	If class imbalance methods were used, state why and how this was done, and any subsequent methods to recalibrate the model or the model predictions	None	None
Fairness	14	D;E	Describe any approaches that were used to address model fairness and their rationale	None	None
Model output	15	D	Specify the output of the prediction model (eg, probabilities, classification). Provide details and rationale for any classification and how the thresholds were identified	page5 Line192-194	Network Details Paragraph 1
Training versus evaluation	16	D;E	Identify any differences between the development and evaluation data in healthcare setting, eligibility criteria, outcome, and predictors	page3 Line102-110	HR-VWI Imaging Method and Image Preprocessing P aragraph1
Ethical approval	17	D;E	Name the institutional research board or ethics committee that approved the study and describe the participant informed consent or the ethics committee waiver of informed consent	page2 Line71-77	Eligibility criteria and patie ntsParagraph1
**Open science**					
Funding	18a	D;E	Give the source of funding and the role of the funders for the present study	page9 Line360-363	[illegible] ph1
Conflicts of interest	18b	D;E	Declare any conflicts of interest and financial disclosures for all authors	page9Line364-366	Acknowledgments Paragra ph2
Protocol	18c	D;E	Indicate where the study protocol can be accessed or state that a protocol was not prepared	None	None
Registration	18d	D;E	Provide registration information for the study, including register name and registration number, or state that the study was not registered	None	None
Data sharing	18e	D;E	Provide details of the availability of the study data	page9Line373-377	[illegible] ph4
Code sharing	18f	D;E	Provide details of the availability of the analytical code§	page9Line373-377	[illegible] ph4
**Patient and public involvement**					
Patient and public involvement	19	D;E	Provide details of any patient and public involvement during the design, conduct, reporting, interpretation, or dissemination of the study or state no involvement	None	None

**Result**					
Participants	20a	D;E	Describe the flow of participants through the study, including the number of participants with and without the outcome and, if applicable, a summary of the follow-up time. A diagram may be helpful	page2-3 Line71-101	Eligibility criteria and patients Paragraph1-3
	20b	D;E	Report the characteristics overall and, where applicable, for each data source or setting, including the key dates, key predictors (including demographics), treatments received, sample size, number of outcome events, follow-up time, and amount of missing data. A table may be helpful. Report any differences across key demographic groups	page6 Line218-226	Basic Information and Plaque Characteristics Paragraph1
	20c	E	For model evaluation, show a comparison with the development data of the distribution of important predictors (demographics, predictors, and outcome)	page6 Line227-243	Predictive performance of Radiomics model and Plaque model Paragraph1
Model development	21	D;E	Specify the number of participants and outcome events in each analysis (eg, for model development, hyperparameter tuning, model evaluation)	page6-7 Line244-271	Deep learning model and Trans-CNN model Paragraph1
Model specification	22	D	Provide details of the full prediction model (eg, formula, code, object, application programming interface) to allow predictions in new individuals and to enable third party evaluation and implementation, including any restrictions to access or reuse (eg, freely available, proprietary)[¶]	page4-5 Line156-197	Network Details Paragraph 1
Model performance	23a	D;E	Report model performance estimates with confidence intervals, including for any key subgroups (eg, sociodemographic). Consider plots to aid presentation	page6 -7 Line217-271	Result Paragraph1-5
	23b	D;E	If examined, report results of any heterogeneity in model performance across clusters. See TRIPOD-Cluster for additional details[‡]	page6 -7 Line257-271	Result Paragraph5
Model updating	24	E	Report the results from any model updating, including the updated model and subsequent performance	page6 -7 Line257-271	Result Paragraph5
**Discussion**					
Interpretation	25	D;E	Give an overall interpretation of the main results, including issues of fairness in the context of the objectives and previous studies	page7 Line273-278	Discussion Paragraph1
Limitations	26	D;E	Discuss any limitations of the study (such as a non-representative sample, sample size, overfitting, missing data) and their effects on any biases, statistical uncertainty, and generalisability	page9 Line343-350	Discussion Paragraph8
Usability of the model in the context of current care	27a	D	Describe how poor quality or unavailable input data (eg, predictor values) should be assessed and handled when implementing the prediction model	None	None
	27b	D	Specify whether users will be required to interact in the handling of the input data or use of the model, and what level of expertise is required of users	None	None
	27c	D;E	Discuss any next steps for future research, with a specific view to applicability and generalisability of the model	page9 Line343-350	Discussion Paragraph8

TRIPOD=Transparent Reporting of a multivariable prediction model for Individual Prognosis Or Diagnosis; AI=artificial intelligence.
* D=items relevant only to the development of a prediction model; E=items relating solely to the evaluation of a prediction model; D;E=items applicable to both the development and evaluation of a prediction model.
† Separately for all model building approaches.
‡ TRIPOD-Cluster is a checklist of reporting recommendations for studies developing or validating models that explicitly account for clustering or explore heterogeneity in model performance (eg, at different hospitals or centres).[1-2]
§ Relates to the analysis code, for example, any data cleaning, feature engineering, model building, and evaluation.
¶ Relates to the code to implement the model to get estimates of risk for a new individual.

1. Debray TPA, Collins GS, Riley RD, et al. Transparent reporting of multivariable prediction models developed or validated using clustered data: TRIPOD-Cluster checklist. BMJ 2023;380:e071018. doi:10.1136/bmj-2022-071018

2. Debray TPA, Collins GS, Riley RD, et al. Transparent reporting of multivariable prediction models developed or validated using clustered data (TRIPOD-Cluster): explanation and elaboration. BMJ 2023;380:e071058. doi:10.1136/bmj-2022-071058

Essential items to include for the reporting of prediction model studies in a journal or conference abstract (TRIPOD+AI for Abstracts*)

Section and item	Item No	Checklist Item	Reported on Page Number/Line Number	Reported on Section/Paragraph
Title	1	Identify the study as developing or evaluating the performance of a multivariable prediction model, the target population, and the outcome to be predicted	Line1-3	Title
Background	2	Provide a brief explanation of the healthcare context and rationale for developing or evaluating the performance of all models	Line6-10	Background
Objectives	3	Specify the study objectives, including whether the study describes model development, evaluation, or both	Line6-10	Background
Methods	4	Describe the sources of data	Line11-12	Methods
	5	Describe the eligibility criteria and setting where the data were collected	Line11-12	Methods
	6	Specify the outcome to be predicted by the model, including time horizon of predictions in case of prognostic models	Line13-18	Methods
	7	Specify the type of model, a summary of the model-building steps, and the method for internal validation†	Line13-18	Methods
	8	Specify the measures used to assess model performance (eg, discrimination, calibration, clinical utility)	Line13-18	Methods

Results	9	Report the number of participants and outcome events	None	None
	10	Summarise the predictors in the final model†	Line19-28	Results
	11	Report model performance estimates (with confidence intervals)	Line19-28	Results
Discussion	12	Give an overall interpretation of the main results	Line19-28	Results
Registration	13	Give the registration number and name of the registry or repository	None	None

TRIPOD=Transparent Reporting of a multivariable prediction model for Individual Prognosis Or Diagnosis; AI=artificial intelligence.

* This checklist is based on the TRIPOD for Abstracts statement published in 2020,[1] but has been revised and updated for consistency with the TRIPOD+AI statement.

† Relevant only to studies describing the development of a prediction model.

1. Moons KGM, Altman DG, Reitsma JB, et al. Transparent Reporting of a multivariable prediction model for Individual Prognosis or Diagnosis (TRIPOD): explanation and elaboration. Ann Intern Med 2015;162:W1-73. doi:10.7326/M14-0698

Article information: https://dx.doi.org/10.21037/qims-24-1723
*As the checklist was provided upon initial submission, the page number/line number reported may be changed due to copyediting and may not be referable in the published version. In this case, the section/paragraph may be used as an alternative reference.
