# Supplementary material for: Can a Generative Artificial Intelligence Model Be Used to Create Mass Casualty Incident Simulation Scenarios? A Feasibility Study
Source: Healthcare (Basel). 2025 Dec 5;13(24):3184. doi: 10.3390/healthcare13243184 (PMC12732669; doi:10.3390/healthcare13243184)
Supplement: Supplementary file 1 [file healthcare-13-03184-s001.zip › Table S1.pdf]

SUPPLEMENTAL TABLE 1: Summary of MCI Simulation Scenarios

Scenarios	Description of Scenario	Number of Patients	Triage Milestones
Scenario 1	Two sedans collide head-on at highway speeds, resulting in multiple patients presenting with severe traumatic injuries.	3	Rapidly assess and prioritize multiple patients presenting after MVC. Evaluate the need for immediate surgical intervention. Coordinate multiple specialties for patient care.
Scenario 2	During a local parade, a car loses control and strikes multiple pedestrians, who present with varying traumatic injuries.	5	Rapid assessment and prioritization of multiple trauma patients following a pedestrian accident. Evaluate the need for immediate surgical intervention. Coordinate care among multiple specialties.
Scenario 3	Two subway cars crash due to a derailment, resulting in multiple traumatic injuries among the passengers of varied severity.	4	Rapid assessment and prioritization of multiple patients after a mass transit accident. Evaluating the need for immediate surgical or interventional radiology procedures. Coordinating care with multiple specialties for optimal patient outcomes.
Scenario 4	A significant explosion in a factory caused by a machinery malfunction results in severe traumatic injuries to several workers.	4	Rapidly assess and prioritize multiple patients presenting after the explosion. Evaluate the extent and depth of burn injuries. Determine the need for

			immediate surgical intervention for shrapnel injuries. Assess and manage inhalation injuries with potential respiratory distress.
Scenario 5	An aged apartment building collapses, trapping several residents, resulting in multiple traumatic injuries of ranging severity.	10	Quick assessment and prioritization of multiple patients post-collapse. Address potential complications from crush injuries. Evaluate immediate needs for surgical intervention. Manage various traumatic injuries in a single setting.
Scenario 6	A shooter opens fire at a shopping center during peak hours, resulting in multiple individuals sustaining gunshot wound injuries of ranging severity.	6	Rapidly assess and prioritize gunshot wound patients based on injury location and severity. Evaluate the need for immediate surgical intervention or imaging. Recognize potential vascular injury and its implications.
Scenario 7	A devastating tornado hits a small town, resulting in extensive destruction. Homes are leveled, with many residents caught in debris, resulting in multiple individuals sustaining traumatic injuries of ranging severity.	10	Rapidly assess and prioritize trauma patients based on the nature and location of injuries. Evaluate the need for surgical intervention, reduction, or stabilization. Manage airway, breathing, and circulation challenges in trauma patients.
Scenario 8	A small plane crashes during takeoff, resulting in fiery wreckage. Immediate first responders pull	4	Rapidly assess and categorize victims based on the severity

	out all passengers on board who have sustained varying degrees of traumatic injury.		and location of injuries. Prioritize trauma management to protect the spine and prevent further damage. Evaluate potential internal injuries from chest and abdominal trauma. Recognize the unique challenges posed by spinal fractures and potential paralysis.
Scenario 9	A fire breaks out in a multi-story hotel, with first responders rescuing multiple guests affected by the incident, with varying degrees of traumatic injury sustained from multiple mechanisms.	8	Quickly assess and categorize victims based on the severity and type of injuries. Prioritize management of smoke inhalation symptoms, ensuring adequate oxygenation. Evaluate and manage second-degree burns, preventing infection and managing pain. Recognize and address potential fractures and internal injuries from falls.
Scenario 10	A boat capsizes on the water near the shore during a routine trip, with first responders rescuing numerous passengers affected by the incident, with multiple passengers identifying with varying degrees of traumatic injury.	8	Quickly assess and categorize victims based on the severity and type of injuries. Prioritize management of near-drowning victims to ensure pulmonary function. Evaluate and manage symptoms of hypothermia, restoring normal body temperature. Recognize and address potential spinal injuries from the capsizing.

Summary of the Mass Casualty Incident (MCI) Simulation Scenarios Opening Descriptions:
