# Supplementary material for: Can a Generative Artificial Intelligence Model Be Used to Create Mass Casualty Incident Simulation Scenarios? A Feasibility Study
Source: Healthcare (Basel). 2025 Dec 5;13(24):3184. doi: 10.3390/healthcare13243184 (PMC12732669; doi:10.3390/healthcare13243184)
Supplement: Supplementary file 1 [file healthcare-13-03184-s001.zip › Table S2.pdf]

SUPPLEMENTAL TABLE 2: Summary of MCI Simulation Scenarios and Supporting Details.

Scenarios	Scenario Summary:	Supporting Materials:	Key Points:
Scenario 1	Three patients were involved in a head-on collision. Bystanders report both cars were at highway speeds.	Simulated patient data: Imaging results (X-rays, FAST exam, CT scans). Simulated trauma bay setup with necessary equipment.	This case highlights the challenges of managing multiple trauma patients simultaneously following a high-impact MVC.
Scenario 2	Five patients struck by a car that lost control during a local parade. Immediate EMS response.	Simulated patient data: Imaging results (X-rays, FAST exam, CT scans). Simulated trauma bay setup with necessary equipment.	This case emphasizes the challenges of managing multiple trauma patients simultaneously following a pedestrian accident.
Scenario 3	Four patients were involved in a subway crash following a derailment. The force of the crash led to severe injuries among multiple passengers.	Simulated patient data: Imaging results (X-rays, FAST exam, CT scans). Simulated trauma bay setup with necessary equipment.	This case underscores the complexities of managing multiple trauma patients in a mass casualty incident resulting from a subway derailment. It challenges the trauma team to prioritize care efficiently and rapidly assess and address life-threatening conditions while ensuring the comprehensive management of all injured patients.
Scenario 4	Workers were operating machinery when there was a sudden explosion, with four patients with severe injuries. Immediate aftermath and injuries are reported by EMS upon arrival.	Simulated patient data: Imaging results (X-rays, FAST exam, CT scans). Simulated trauma bay setup with necessary equipment, including burn care supplies and equipment, along with airway cart.	This case presents a machinery malfunction leading to an explosion at a factory exposes workers to multi-system trauma, challenging the emergency team's comprehensive care capabilities.
Scenario 5	The ten patients were in their apartments when the building collapsed. Rescue efforts brought them out from the debris, some with delayed extrication.	Simulated patient data: Imaging results (X-rays, FAST exam, CT scans). Simulated trauma bay setup with necessary equipment.	This case stresses the unique challenges posed by building collapses, requiring swift actions, multitasking, and collaboration.
Scenario 6	Six patients were shopping when an	Simulated patient data: Imaging results (X-	This scenario underscores the

	active shooter opened fire. Immediate medical response was initiated at the scene, and patients were transferred rapidly to the hospital.	rays, FAST exam, CT scans). Simulated trauma bay setup with necessary equipment, including tourniquet.	complexities and urgencies of treating multiple gunshot wound victims simultaneously in an emergency setting. Quick assessment, prioritization, and management can significantly impact outcomes, emphasizing the importance of streamlined communication and efficient trauma protocols.
Scenario 7	Ten patients were inside or near their homes when the tornado struck. Immediate medical response was initiated at the scene, and patients were rapidly transferred to the hospital.	Simulated patient data: Imaging results (X-rays, FAST exam, CT scans). Simulated trauma bay setup with necessary equipment.	This scenario highlights the challenges of managing diverse injuries stemming from a natural disaster. Effective triage, assessment, and rapid intervention can greatly influence outcomes.
Scenario 8	The four patients were onboard a small airplane during takeoff when the crash occurred. Bystanders noted a fiery explosion upon impact. Immediate medical response was dispatched to the scene, and all passengers were extricated and rapidly transferred to the hospital.	Simulated patient data: vitals, initial findings, consciousness levels. Imaging results: Head CT scans, chest X-rays, FAST exam (ultrasound), MRI for spinal cord assessment. Simulated trauma bay setup with essential equipment and burn care resources.	This scenario emphasizes the intricacies of managing trauma resulting from a plane crash, which can lead to a mix of injuries. The team's efficiency in triaging, assessing, and treating each patient can greatly influence outcomes.
Scenario 9	The eight patients were guests at a multi-story hotel when a fire broke out. Due to the rapid spread of the fire, they sustained various injuries including smoke inhalation, burns, and injuries from jumping off the building to escape. Immediate medical response was	Simulated patient data: vitals, initial findings, consciousness levels. Imaging results: Chest X-rays for smoke inhalation assessment, X-rays for fractures, FAST for internal injury assessment; Simulated emergency room setup with essential equipment, oxygen support, and	The scenario underlines the complexities of managing trauma from a plane crash. The team's effectiveness in triaging, assessing, and treating each patient significantly influences outcomes.

	dispatched to the scene and all patients were rapidly transferred to the hospital.	care resources, including airway cart.	
Scenario 10	The eight patients were passengers on a ferry when it capsized near the coast. Due to the sudden capsizing of the ferry, they sustained various injuries including drowning, hypothermia, spinal injuries, and complications from near-drowning. Immediate medical response was dispatched to the scene and all patients were rapidly transferred to the hospital.	Simulated patient data: vitals, initial findings, consciousness levels. Imaging results: Chest X-rays for lung injury assessment, X-rays for fractures, FAST ultrasound for internal thoracoabdominal injury assessment, Simulated emergency room setup with essential equipment, oxygen support, and care resources. Rewarming equipment.	This scenario showcases the challenges of managing victims of a boating accident, where a myriad of injuries from various mechanisms present simultaneously. The team's efficiency in triaging, evaluating, and treating each patient, while juggling the diverse clinical presentations, plays a crucial role in determining patient outcomes.

Summary of the Mass Casualty Incident (MCI) Simulation Scenarios Supporting Details
