# Supplementary material for: Can a Generative Artificial Intelligence Model Be Used to Create Mass Casualty Incident Simulation Scenarios? A Feasibility Study
Source: Healthcare (Basel). 2025 Dec 5;13(24):3184. doi: 10.3390/healthcare13243184 (PMC12732669; doi:10.3390/healthcare13243184)
Supplement: Supplementary file 1 [file healthcare-13-03184-s001.zip › Table S3.pdf]

SUPPLEMENTAL TABLE 3: Summary of MCI Simulation Scenarios Scoring Breakdown.

Scenarios	Final SSET Scores	Domain 1	Domain 2	Domain 3	Domain 4	Domain 5	Domain 6	Review Time (mins)
Scenario 1	93	34	9	14.5	19	8.5	8	35
Scenario 2	92.5	34	9	14	19	8.5	8	54.5
Scenario 3	93	34	9	14	19	9	8	22.5
Scenario 4	94	33	10	14.5	19.5	9	8	18.5
Scenario 5	90	32	9.5	13.5	19	8.5	7.5	21
Scenario 6	96	33.5	10	15	20	9	8.5	25
Scenario 7	94	34	10	14	19	9	8	25
Scenario 8	96	34	10	15	19.5	9	8.5	26
Scenario 9	95	34	10	15	19	9	8	21.5
Scenario 10	96	34	10	15	19.5	9	8.5	26.5

Summary of the Mass Casualty Incident (MCI) Simulation Scenarios SSET Score Breakdown and Review Time:
