# Supplementary material for: Can a Generative Artificial Intelligence Model Be Used to Create Mass Casualty Incident Simulation Scenarios? A Feasibility Study
Source: Healthcare (Basel). 2025 Dec 5;13(24):3184. doi: 10.3390/healthcare13243184 (PMC12732669; doi:10.3390/healthcare13243184)
Supplement: Supplementary file 1 [file healthcare-13-03184-s001.zip › Table S4.pdf]

SUPPLEMENTAL TABLE 4: Summary of MCI Simulation Scenarios Databases.

Database	Maintaining Agency	Focus	Data Contents	Number of Cases
**National Trauma Data Bank (NTDB)**	American College of Surgeons (ACS)	Hospital-based trauma registry	Patients treated at participating trauma centers (Level I–III primarily)	~130,000 cases annually
**National Electronic Injury Surveillance System (NEISS)**	U.S. Consumer Product Safety Commission (CPSC)	Consumer product–related injuries	Probability sample of U.S. emergency departments	~500,000 ED cases (NEISS-AIP) annually
**National Fire Protection Association (NFPA) Fire Incident Data**	NFPA (nonprofit)	Fire incidents, fire-related injuries & fatalities	U.S. fire departments (sampled or full participation depending on dataset)	~1.3 million cases annually
**U.S. Coast Guard (USCG) Marine Casualty & Incident Database**	United States Coast Guard	Maritime injuries, boating accidents, search-and-rescue outcomes	Commercial and recreational maritime incidents in U.S. waters	~4,000 cases annually
**National Transportation Safety Board (NTSB) Accident Database**	NTSB (independent federal agency)	Major transportation accidents (aviation, highway, rail, pipeline, marine)	National-level events that meet NTSB investigation thresholds	~1,000 aviation accident cases annually

Summary of the Mass Casualty Incident (MCI) Simulation Scenarios Databases
