# Supplementary figures and images for: Can a Generative Artificial Intelligence Model Be Used to Create Mass Casualty Incident Simulation Scenarios? A Feasibility Study
Source: Healthcare (Basel). 2025 Dec 5;13(24):3184. doi: 10.3390/healthcare13243184 (PMC12732669; doi:10.3390/healthcare13243184)

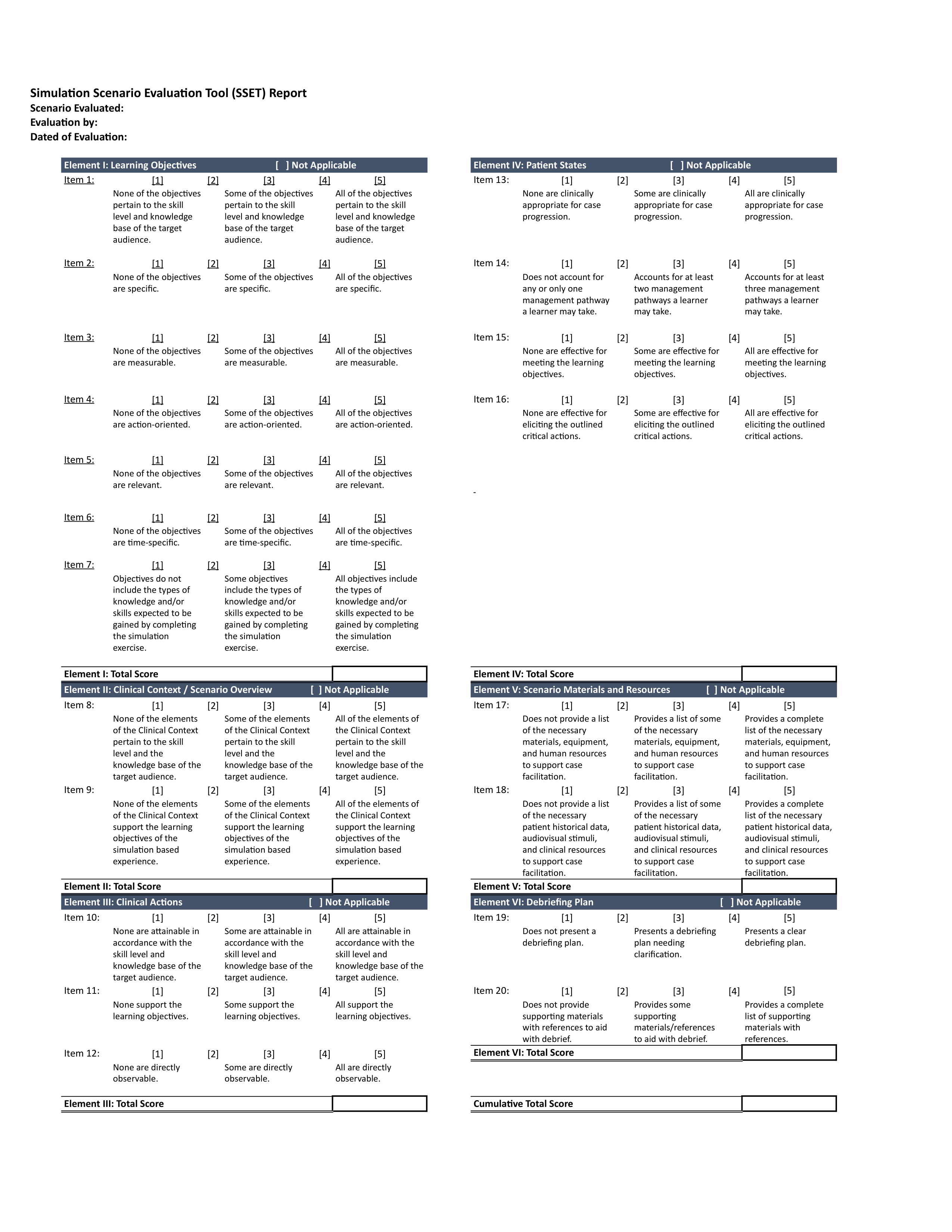

Supplement: Supplementary file 1 [file healthcare-13-03184-s001.zip › Figure S1.png]
